# Humeral Supracondylar Fractures in Children: A Novel Technique of Lateral External Fixation and Kirschner Wiring

**DOI:** 10.5704/MOJ.1607.008

**Published:** 2016-07

**Authors:** RY Kow, AR Zamri, JK Ruben, S Jamaluddin, MT Mohd-Nazir

**Affiliations:** Hospital Kuala Lipis, Kuala Lipis, Pahang, Malaysia

**Keywords:** Supracondylar fracture, humerus, external fixator, Kirschner wire

## Abstract

**Introduction:** Supracondylar fracture of the humerus is the most common fracture around the elbow in children. Pinning with Kirschner wires (K-wires) after open or closed reduction is generally accepted as the primary treatment modality. However, it comes with the risk of persistent instability and if the K-wire is not inserted properly, it may cause displacement and varus deformity. We present our two-year experience with a new technique of lateral external fixation and K-wiring of the humeral supracondylar fracture.

**Materials and Methods:** A total of seven children with irreducible Gartland Type III supracondylar humeral fracture were treated with closed reduction and lateral external fixation and lateral Kirschner wiring. Patients with ipsilateral radial or ulnar fracture, open fracture and presence of neurovascular impairment pre-operatively were excluded. All the patients were followed up at one, three and six weeks and three and six months. The final outcomes were assessed based on Flynn’s criteria.

**Results:** All the patients achieved satisfactory outcomes in terms of cosmetic and functional aspects. All patients except one (85.5%) regained excellent and good cosmetic and functional status. One patient (14.3%) sustained pin site infection which resolved with oral antibiotic (Checketts- Otterburn grade 2). There was no neurological deficit involving the ulnar nerve and radial nerve.

**Conclusion:** The introduction of lateral external fixation and lateral percutaneous pinning provide a promising alternative method for the treatment of humeral supracondylar fracture. This study demonstrates that it has satisfactory cosmetic and functional outcomes with no increased risk of complications compared to percutaneous pinning.

## Introduction

Supracondylar fracture of the humerus is the most common fracture around the elbow in children^1,2^. It accounts for 17% of all limb fractures in children^3,4^. Severely displaced humeral supracondylar fracture Gartland III carries the risks of neurovascular injury and compartment syndrome^5^. There is still no consensus on the treatment of displaced humeral supracondylar fractures in children and it is based on the preference and experience of the treating surgeons. For many years, surgeons have been using the Dunlop traction or cast immobilization after closed manipulative reduction but studies show that these are associated with complications of malunion producing varus and valgus deformities and elbow stiffness with potential to compromise neurovascular structures^6-9^.

Nowadays, pinning with Kirschner wires (K-wires) after open or closed reduction is generally accepted as the primary treatment modality^8,10^. Various methods of pinning have been described, including medial, lateral, combined medial and lateral, posterior and anteromedial approaches, with each having its own pros and cons^11^. However, pinning with K-wires comes with the risk of persistent instability and if the K-wire is not inserted properly, it may cause displacement and varus deformity^12^. Besides that, the splint or cast applied after K-wiring prevents early mobilization and subsequently compromise the functional elbow recovery^12^.

A new technique of lateral external fixation of the humeral supracondylar fracture has been described to overcome the problems arising from pinning of the humeral supracondylar fracture^12^. Using this method as a guide, we modified this technique so that a systematic approach can be described with minimal interpersonal discrepancy between surgeons. We present our two-year experience with this surgical approach.

## Materials and Methods

This is a retrospective study conducted in patients treated for supracondylar humeral fractures in a single institution from September 2013 to September 2015. A total of 7 children with Gartland Type III supracondylar humeral fractures were treated with closed reduction and lateral external fixation and lateral Kirschner wiring. In this study, we reviewed all the patients to identify the demographic data (age, gender, mechanism of injury), site of injury, type of fracture, operative time, days of hospitalization, post-operative complications (pin tract infection, nerve palsy) and parents’ satisfaction. Functional and cosmetic outcomes were assessed at the final follow-up examinations at six months post-operation based on the Flynn’s criteria which evaluate loss of motion, carrying angle and malalignment.

Patients included in this study met the following criteria: 1) Unilateral type III supracondylar humeral fracture; 2) Irreducible with closed method. Patients were excluded from this study based on the following criteria: 1) Ipsilateral radial or ulnar fracture; 2) open fracture; 3) presence of neurovascular impairment pre-operatively.

### Surgical Technique

**Fig. 1:**
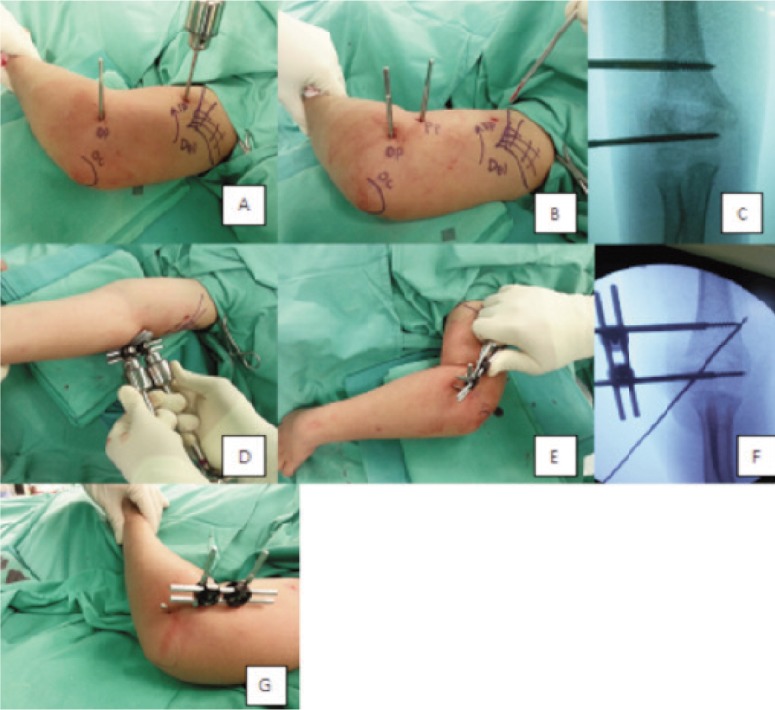
Show intra-operative photographs of the patient. **(A)** The first 3.5 mm Schanz pin was inserted at the distal fragment at the metaphysis (DP) and a temporary 4 mm Schanz pin (TP) was inserted at the proximal 1/3rd of the humerus, just distal to the insertion site of the deltoid tendon(Del). Acting as a joystick, the temporary Schanz pin can be internally or externally rotated to correct any rotational deformity of the proximal fragment. **(B)** TP was removed after a proximal Schanz pin (PP) was inserted, parallel to the TP. **(C)** location of the PP and DP in the imaging intensifier monitor. **(D)** Acute distraction of 1-3mm was done using the T-handle bar to correct the coronal deformity. **(E)** A retractor was used to correct the sagittal deformity. **(F)** A cross K-wire was inserted to maintain the reduction. **(G)** Range of motion was checked at the end of the surgery.

All procedures were performed under general anaesthesia. Surgery was performed by the same orthopaedic surgeon to prevent interpersonal discrepancy. Patient was positioned supine with the affected upper limb at the edge of the bed. The affected elbow was rested on the receiving end of the image intensifier, which served as the work table. All patients had two 3.5 mm Schanz pins inserted at the proximal and distal parts of the fracture respectively. All but one patient had one 1.8 mm Kirschner wire inserted laterally to further stabilize the fracture. One patient had two lateral 1.8 mm Kirschner wires inserted because of severely displaced and comminuted fracture (Figure 1A-G).

**Fig. 2:**
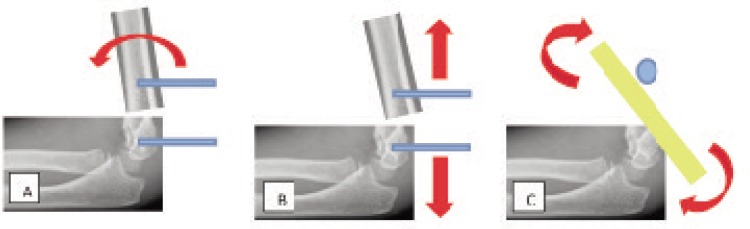
Shows the schematic diagram of the surgery. **(A)** temporary Schanz pin (blue bar) was used to correct the rotational deformity. **(B)** Acute distraction of 1-3mm to correct the coronal deformity. **(C)** A retractor (yellow) was used to correct the sagittal deformity.

The first 3.5 mm Schanz pin was inserted at the distal fragment at the metaphysis, just above the growth plate (Figure 1A & B). A temporary 4 mm Schanz pin was inserted at the proximal third of the humerus, just distal to the insertion site of the deltoid tendon (Figure 1A). Acting as a joystick, the temporary Schanz pin could be internally or externally rotated to correct any rotational deformity of the proximal fragment (Figure 2A). Next, after the rotational deformity was corrected, the second 3.5 mm Schanz pin was inserted 1 cm proximal to the fracture line, parallel to the temporary Schanz pin (Figure 1B-D). The second Schanz pin was inserted under direct vision after a stab incision with a size 11 blade. This was to prevent injury to the radial nerve which crosses the lateral supracondylar ridge of the humerus at the diaphyseal-metaphyseal junction before it pierces the lateral intermuscular septum to enter the anterior compartment of the arm^13^. The temporary Schanz pin was then removed (Figure 1C).

Rods and clamps were attached to both proximal and distal Schanz pin but they were not tightened. Next, two T-handle bars were attached to both the Schanz pins just above the clamps (Figure 1E). The two 3.5 mm Schanz pins acted as joysticks with which the surgeon could manipulate both proximal and distal fragments to achieve coronal reduction of the fracture by acute distraction of 1-3mm (Figure 2B). Once a desired reduction was achieved, the clamps were tightened to maintain the reduction. A retractor was then used as a leveller in between the two Schanz pins to correct the coronal deformity (Figures 1F & 2C). One 1.8 mm Kirschner wire was inserted laterally, crossing in between two Schanz pins to hold stabilise the fracture and it acted as an anti-rotational device before final tightening of the clamps (Figure 1G). The final check involved testing the range of motion of the elbow and fracture stability under image intensifier before dressing (Figure 2A-C).

**Fig. 3:**
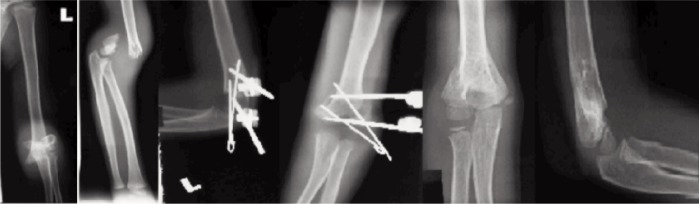
Serial radiographs of a left humeral supracondylar fracture Gartland type III successfully treated with lateral external fixation and Kirschner wiring.

**Fig. 4:**
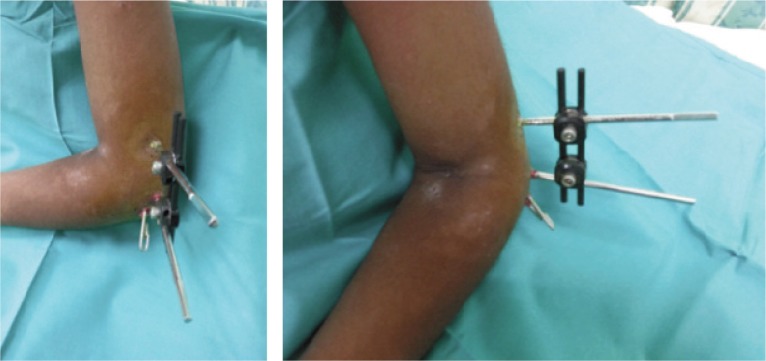
Left elbow of a patient three weeks post-operative shows a combination of external fixator and Kirschner wire providing a stable fixation of the fracture site.

After pin site dressing, an arm sling was applied for patients’ comfort. Parents were educated regarding the importance of pin site dressing to prevent pin site infection. Patients were discharged home on post-operative Day 2 after wound inspection. Immediately post-operation, patients were allowed active range of motion exercises of the affected limb as tolerated. Patients were reviewed at one week postoperatively to inspect the wound. The Kirschner wire was removed at three weeks post-operatively and the external fixator was removed at six weeks post-operatively. All patients were followed up for a duration of three months to one year. (Figure 3,4).

## Results

A total of seven patients (six males and one female) were included in this study. All the patients were between 5 and 12 years of age, with a mean age of 7.8 years (Table I). All the patients were right hand dominant. The majority of the patients had their non-dominant upper limbs injured (71%). Most of the patients had sustained supracondylar fractures after falls (85.8%) with only one patient with injury during sports activity (14.2%) (Table II). All the patients had surgical fixation of the fractures between one and six days post-trauma with a mean of four days. The mean operation duration was 50 minutes (range: 30 to 76 minutes). All patients were discharged within three post-operative days (Table III).

**Table I:** Shows the demographic data of the patients

Demographic data Age (years)	Mean 7.8 (range 5-12)	Number of patients	Percentage
Gender	Male	6	86%
	Female	1	14%
Side of fracture	Right	2	29%
	Left	5	71%
Type of fracture	Gartland type III	7	100%

**Table II:** Shows the mechanism of injury

Mechanism of injury	Number of patients	Percentage
Fall from bicycle	2	28.6%
Fall from height	2	28.6%
Fall while playing	2	28.6%
Sports injury	1	14.2%

**Table III:** Shows the hospitalization details

Hospitalization details	Mean	Range
Days of hospitalization prior to surgery	4	1-6
Surgery duration (minutes)	50	30-76
Post-operative stay (day)	2	1-3

The patients were reviewed within the first week postoperatively to look for any signs of pin site infection and at three weeks to remove K-wires. The external fixators were removed at six weeks post-operatively. They were followed up for a duration of three months up to six months. During our final assessments, all patients had achieved fracture union. The treatment outcomes were assessed using the Flynn’s criteria, which assessed the carrying angle and range of motion of the affected elbow^14^ (Table IV). The normal range of motion of the elbow is defined as flexion of 140 degree to 150 degree with extension to 0 degree or even slight hyperextension^14^. All the patients achieved satisfactory outcomes in terms of cosmetic and functional aspects (Table IV). In the term of cosmetic factor, four (57.1%) patients had excellent outcomes while two (28.6%) patients had good outcomes and one (14.3%) patient fair outcome. In terms of functional factor, five (71.4%) of the patients had excellent outcomes while two (28.6%) had good outcomes. One patient (14.3%) sustained pin site infection which resolved with oral antibiotics (Checketts-Otterburn grade 2)^15^. There was no neurological deficit involving the ulnar nerve and radial nerve. All parents were satisfied with the treatment outcomes.

**Table IV:** Shows the final outcome based on Flynn’s criteria

Final Results		Cosmetic factor: Loss of carrying angle (degree)	Number of Patients (%)	Functional Factor: Loss of motion (degree)	Number of Patients (%)
Satisfactory	Excellent	0-5	4 (57.1%)	0-5	5 (71.4%)
	Good	6-10	2 (28.6%)	6-10	2 (28.6%)
	Fair	11-15	1 (14.3%)	11-15	0
Unsatisfactory	Poor	>15	0	>15	0

## Discussion

In our series, the mean age of the patients is was 7.8 years, which corresponded to other studies with a mean age of 6 to 8.9 years^1,8,10^. Similarly, boys were more prone to supracondylar fractures as compared to girls and the non-dominant upper limbs more frequently involved^1^.

Some recent reports claim that delay in definitive treatment of type III humeral supracondylar fractures did not affect the outcomes but there are some studies that recommend treatment as early as possible^16-18^. In our series, all patients underwent the definitive surgical fixation electively within one to six days hospitalization. We attempted preliminary fracture reduction with conventional closed manipulative method before proceeding with external fixation and K-wiring, thus explaining the long operating time (mean of 50 minutes). By using this technique, acceptable reductions could be achieved in all patients without the need to proceed to open reduction.

At the treatment end point, all patients achieved fracture unions with satisfactory cosmetic and functional outcomes based on the Flynn’s criteria^14^. The greatest advantage of using this technique is that open reduction could be avoided in those patients with severely displaced supracondylar humeral fractures. This is accompanied by smaller wounds and thus reduced pain. In children, pain is a preventive factor for early mobilization, which then indirectly affects the functional outcomes. On top of that, external fixation and K-wiring provide rigid fracture fixation, thus allowing early mobilization of the elbow compared to K-wiring alone which normally requires a splint or a back slab post-operatively. The rigid fixation provided by the external fixator and K-wires prevents displacement of the distal fracture fragment which may result in varus or valgus deformity^12^.

**Fig. 5:**
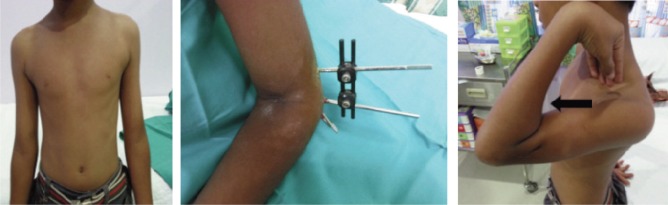
The patient with good results, both functionally and cosmetically six months post-operatively. The pin site scars were barely visible (black arrows).

In this method, we avoided inserting a K-wire medially, hence the risk of iatrogenic ulnar nerve palsy could be reduced^19^. However, extra care should be taken during the placement of the proximal Schanz pin due to the close proximity to the radial nerve^13^. In our series, we made a mini incision for placement of the proximal Schanz pin under direct vision to avoid any iatrogenic radial nerve injury. All proximal Schanz pin were inserted within 2 cm from the fracture line under direct vision.

Pin tract infection rates in patients undergoing percutaneous K-wiring are between 5.9% to 19.7% based on previously published studies^20-23^. Only one of our patients (14%) developed pin site infection which resolved with one week of oral antibiotic (Checketts-Otterburn grade 2) without other sequelae^15^. The patient was a boy who lived in a rural area and was not compliant to pin site dressing as counselled due to poor family educational level and social support.

The technique of lateral external fixation of humeral supracondylar fractures in children has been reported previously; however we may claim to be the first to provide a step-by-step approach in terms of correcting the rotational, sagittal and coronal deformity of the fracture. By using this as a guide, junior surgeons may have a smoother learning curve in terms of managing displaced humeral supracondylar fractures.

The limitations of this study are that it has a small sample size, it is a retrospective study, and there is no control group. Nevertheless, the review of the cases in this study over a two-year period demonstrates that lateral external fixation and K-wiring is an alternative method to percutaneous pinning alone, with promising results.

## Conclusion

Displaced supracondylar humeral fracture remains a challenge to orthopaedic surgeons. The introduction of lateral external fixation and lateral percutaneous pinning provides a promising alternative method for the treatment of this type of fracture. This study demonstrates that it has satisfactory cosmetic and functional outcomes with no increased risk of complications compared to percutaneous pinning.
